# Female reproductive fluids ‘rescue’ sperm from phenotypic ageing in an external fertilizer

**DOI:** 10.1098/rspb.2023.0574

**Published:** 2023-05-31

**Authors:** Jessica H. Hadlow, Jonathan P. Evans, Rowan A. Lymbery

**Affiliations:** Centre for Evolutionary Biology, School of Biological Sciences, University of Western Australia, Crawley, WA, Australia

**Keywords:** sperm longevity, postcopulatory sexual selection, phenotypic plasticity, chemoattractants, sperm velocity-longevity trade-off, sperm senescence

## Abstract

Female reproductive fluids (FRFs) serve key reproductive functions in sexually reproducing animals, including modifying the way sperm swim and detect eggs, and influencing sperm lifespan. Despite the central role of FRF during fertilization, we know surprisingly little about sperm–FRF interactions under different environmental conditions. Theory suggests that in external fertilizers FRF may ‘rescue’ sperm from ageing effects as they search to fertilize eggs. Here, we test the interaction between these two fundamental properties of the fertilization environment, ejaculate age (i.e. time since ejaculation) and FRF, on a range of functional sperm phenotypes in a broadcast spawning mussel, *Mytilus galloprovincialis*. We found that the effects of ejaculate age on multivariate sperm motility traits and total sperm motility were altered by FRF, and that longer-lived sperm exhibit stronger, likely more advantageous, responses to FRF after periods of ageing. We also detected significant among-male variation in the relationship between sperm motility traits and ejaculate age; notably, these patterns were only revealed when sperm encountered FRF. Collectively these findings underscore the importance of considering female reproductive physiology when interpreting ageing-related declines in sperm motility, as doing so may expose importance sources of variation in sperm phenotypic plasticity among males and environments.

## 1. Introduction

Sperm exhibit substantial phenotypic plasticity as a result of the variable post-ejaculatory environments they experience [1,2], where they undergo behavioural, structural and/or molecular changes in preparation for fertilization [3]. In internal fertilizers, for example, sperm encounter complex female reproductive tracts that require phenotypic adjustments to enable prolonged storage in specialized organs, or effective interactions with the reproductive tract epithelium (reviewed in [3–5]). Externally fertilizing sperm also undergo structural or behavioural changes induced by the female environment (e.g. eggs and their surrounding fluids) prior to fertilization, while additionally responding to variable physical and chemical conditions outside the homeostatic protection of reproductive tracts [6]. Despite the central role that phenotypic changes to sperm play in the fertilization process, we know little about the extent of such changes across multiple, and potentially interacting, components of the fertilization environment (but see [7–9]).

Female reproductive fluids (i.e. fluids derived from female reproductive tracts or eggs; FRF) serve key functions in most sexually reproducing animals [10] and are known to alter sperm viability [11–16], sperm physiology [17,18] and sperm motility [13,19–23]. Furthermore, gradients of FRF stimulate changes in sperm velocity, path trajectory, flagella waveforms and subsequently, directional orientation toward eggs (e.g. sperm chemokinesis and chemotaxis; [24–27]). However, while FRF is known to play an integral role in mediating sperm behaviour in the fertilization environment, there have been few tests of whether FRF interacts with other environmental factors that affect sperm phenotypes (but see [15]).

The sperm of both external and internal fertilizers can experience considerable time lags between ejaculation and encountering eggs. Sperm cells have limited energetic resources [28] and often exhibit changes in behaviour with time after ejaculate release that can have negative fitness consequences, e.g. declines in velocity over time [29–31]. For the purposes of this study, we consider the time following ejaculate release as ‘ejaculate age’ and the temporal changes in sperm motility traits to be reflective of ‘sperm longevity’. When there is a prolonged lag between ejaculate release and sperm–egg encounters, it may be advantageous for sperm to conserve energy to ensure they can respond to female/egg cues when required. Indeed, there is evidence that sperm longevity trades off with initial swimming velocity [32,33], and that slow sperm may have a competitive advantage when they must swim for extended periods before fertilization [32,34,35]. The evidence for such trade-offs, however, remains equivocal in many systems [36–38]. One possible, but as yet untested, explanation for this ambiguity is that velocity–longevity trade-offs are masked or altered in the presence of FRF, which are known to affect sperm lifespans (e.g. [12,19,20,23,39]).

Sessile marine broadcast spawners are ideal, yet under-used, systems for disentangling interactive effects of ejaculate age and FRF on sperm traits. Broadcast spawning, defined as gamete release from both sexes into external environments, was an important early transition in the evolution of mating systems and remains the predominant reproductive strategy in marine organisms [40–42]. In broadcast spawners, as in many other taxa, unfertilized eggs release FRF in the form of chemoattractants that act as chemical ‘signposts’ to guide sperm towards eggs, typically via increases in velocity and path linearity, and altered flagella beat patterns [6,25–27]. While sperm–egg encounters can occur soon after gamete release in some synchronous spawners, fluctuating conditions during spawning events (e.g. water flow, tidal movements) and variation in the distribution and density of spawning individuals often result in sperm limitation (too few sperm to fertilize a given set of eggs; [43]) and delayed gamete encounters [44–46]. Therefore, sperm are often suspended in the water column for prolonged periods before eggs and associated FRF are locally available. In these contexts, sperm that can conserve energy might be better at ‘searching’ for and responding to egg-derived FRF [47,48]. The interval between ejaculate release and sperm–FRF encounters in these systems offers a valuable opportunity to explore the effects of FRF on aged sperm because, unlike in internal fertilizers, fertilization is dependent on sperm behaviour both before and after encountering female fluids in the external environment [6,10].

Here, we use the broadcast spawning mussel, *Mytilus galloprovincialis*, and a repeated-measures experimental design to test the interactive effects of ejaculate age and egg-derived chemoattractants (hereafter, FRF) on sperm motility traits. *M. galloprovincialis* occupies habitats where environmental fluctuations generate variation in sperm–egg encounter rates during spawning [44,49]. In this species, sperm traits, and patterns of selection upon them, are altered by the presence of FRF [50], and slower-swimming sperm are favoured in conditions where sperm–egg encounters are delayed [48,51]. The ‘searching’ behaviour of sperm in this species is characterized by slow, curved swimming paths, often with large head movements, that allow sperm to detect FRF gradients (e.g. [48,50]). Upon encountering FRF, sperm velocity and path linearity typically increase, as is characteristic of chemotactic responses (e.g. [21,52]). Recent work in the closely related *M. edulis* reported that sperm use different metabolic strategies in seawater compared to egg-derived FRF [18], suggesting that sperm energy consumption, and thus the relationship between sperm motility and longevity, may be influenced by FRF. Accordingly, we test for an interaction between FRF and ejaculate age on sperm velocity, path trajectory, flagella beat patterns and sperm head movements. We expect that FRF will enhance the longevity of sperm motility, as reported in other species (e.g. [19,23,32,39]). We also test the novel prediction that among-individual variation in sperm motility phenotypes will change over time and in response to FRF. Finally, we test for the presence of a trade-off between sperm longevity and sperm energetic expenditure (motility) in seawater, and whether sperm longevity in seawater determines the capacity of sperm to respond to FRF at different ages [47,48]. Here, we predict sperm that are longer-lived in seawater will be capable of stronger responses to FRF after periods of ageing, e.g. increased velocity and linearity, than shorter-lived sperm.

## 2. Material and methods

### (a) Study organism and gamete collection

The blue mussel, *M. galloprovincialis,* inhabits temperate intertidal and subtidal zones in the Northern and Southern hemispheres [53]. We collected specimens from Woodman Point, Western Australia (32°14′03.6″S, 115°76′25″E) from June to September 2020. We followed standard protocols for spawning, adjusting gamete concentrations and collecting egg water—seawater containing egg-derived FRF ([21,48,54,55]; see electronic supplementary material). Additionally, we followed freeze–thaw protocols to store and use egg water (FRF), which do not affect its bioactivity ([17]; see electronic supplementary material, table S1). Prior to experimental assays, sperm samples were adjusted to a working concentration of 4 × 10^6^ sperm ml^−1^ and eggs samples were adjusted to 5 × 10^4^ eggs ml^−1^ [21,50–52].

### (b) Experimental design

We tested whether the presence or absence of FRF mediates any observed effect of ejaculate age on sperm motility traits known to predict reproductive fitness (e.g. [21]; and see §2d below). Further, our repeated-measures design enabled us to determine whether FRF influences among-male variation in the relationship between ejaculate age and sperm motility. We use the term ejaculate age here because our ageing treatment is applied at the ejaculate level. For logistical reasons, our experiment comprised a series of blocks, in which ejaculates were aged in seawater, and then we repeatedly tested sperm motility at different ages in either seawater or in egg-derived FRF (see below). Sperm show signs of ‘ageing’ when diluted, e.g. changes in motility or fertilization capacity, a phenomenon known as the respiratory dilution effect [45,56]. Therefore, diluting the ejaculates prior to motility assays allows us to detect ageing effects. Each ejaculate was first tested immediately after dilution, with three subsequent repeats occurring approximately 1 h apart (i.e. tests occurred at approximately 0, 1, 2 and 3 h post-dilution; see electronic supplementary material, figure S1). At each timepoint, subsamples of ageing ejaculate were added to (i) a seawater control and (ii) FRF (pooled from multiple females) for motility assays. Each male's ejaculate was therefore assayed eight times: in two environmental treatments and at four ages. Each block consisted of ejaculates from 5 to 7 replicate males, and a pool of thawed FRF (egg water) from 5 to 6 females. We used pooled FRF to control for among-female variation in the quality of eggs and their associated fluids, and to control for male-by-female interaction effects that occur in *M. galloprovincialis* [21,54,57]. We performed seven experimental blocks and obtained a total sample size of *n* = 43 males tested across the different environment and age combinations (344 assays in total).

### (c) Sperm motility

We analysed sperm motility in seawater or egg-derived FRF using computer-assisted sperm analysis (CASA; CEROS II, Hamilton Thorne, Beverly, MA, USA). An hour prior to the first motility analyses, frozen FRF samples from the females in each block were thawed and stored at 4°C until required, at which point the samples were brought to ambient room temperature (22°C). FRF from each female was mixed in equal portions before the commencement of CASA. Seawater was also kept at 4°C and brought to room temperature when required. Just prior to the first motility assays, we used seawater to dilute eight ejaculate subsamples from each male to the working concentration described above and thereafter allowed the samples to age in seawater at that concentration.

For each set of motility analyses, we mixed 50 µl of the diluted, ageing ejaculate sample with 50 µl of either seawater or FRF. We then added 4 µl of mixed sample to a 12-well multi-test slide previously coated in 1% polyvinyl alcohol to prevent sperm from sticking to the slide [58]. We immediately analysed sperm motility using CASA. To account for differences between male spawning time and the time of dilution, we recorded ‘ejaculate age’ as minutes since spawning. To avoid any systematic bias in measurement order, we alternated whether we first assayed sperm in FRF or seawater at each set of analyses and for each male (electronic supplementary material, figure S1). For each sample, we ensured that CASA videos were haphazardly chosen as the first fields of view encountered that contained sufficient numbers of sperm cells.

All ejaculates within a block were diluted at the same time, and the first motility analyses were conducted 222.1 ± 3.5 min after spawning on average (± s.e.). Prior to dilution, ejaculates had been kept at very high concentrations (in the order of 10^8^ sperm ml^−1^) in 20 ml of seawater in the spawning cups to minimize respiratory dilution effects [45,56,59]. Hence, the exact time at which the first assays were conducted was not expected to have a major effect on our ability to assess temporal patterns in sperm motility. The average time between the two assays from the same male within a timepoint (i.e. one in FRF and one in seawater) was 5.5 ± 0.2 min (mean ± s.e.). We tracked an average of 179.23 ± 2.24 (mean ± s.e.) motile sperm per assay across all treatment and age combinations. Static cells were determined by threshold values of 4 µm s^−1^ for straight-line velocity (VSL) and 19.9 µm s^−1^ for average path velocity (VAP; [21,48]).

### (d) Statistical analyses

#### (i) Principal component analysis on sperm motility traits

CASA generates multiple correlated measures of sperm motility (see electronic supplementary material, table S2 for correlation matrices of sperm traits in each treatment). As it is widely recognized that selection acts on sperm trait combinations (e.g. correlational selection), and that ejaculate and sperm components are functionally integrated [48,50,52,60–63], we used principal component analysis (PCA) to reduce these variables and avoid multiple testing of correlated parameters, using the package ‘FactoMineR’ [64] in R v.4.1.1 [65].

In our analysis of sperm motility, we included three measures of sperm velocity (curvilinear velocity, VCL; average path velocity, VAP; and straight-line velocity, VSL), three measures of path straightness (linearity, LIN; straightness, STR; wobble, WOB), a measure of sperm head movement (amplitude of lateral head movement from the average path, ALH) and flagella beat frequency (the frequency with which the curvilinear path crosses the average path, BCF) as parameters in a PCA. This analysis yielded two principal components (PCs) with eigenvectors > 1 (PC1 and PC2; table 1; [66]), which were loaded in a similar manner to PCs of sperm motility that predict fertilization success in *M. galloprovincialis* ([21]; see §3 for trait loadings). Furthermore, published work in mussels has consistently demonstrated that selection targets sperm linearity and velocity as multivariate traits. Specifically, sperm with slow, curved swimming trajectories have greater fertilization success in seawater, and sperm with faster, straighter swimming perform better in egg-derived FRF [48,50–52]. Therefore, in our subsequent analyses, we treat PC1 and PC2 as composite motility traits described by their major loadings (loadings of 0.3 or greater were considered as major contributors to the axis [66]). Additionally, we repeated our main analyses (described below) on a subset of the raw sperm motility parameters that loaded strongly on the two PCs in this study: VCL, LIN, ALH and BCF. The ancillary results were qualitatively similar to those reported in the main text and support the broad conclusions of our analyses (see electronic supplementary material).

**Table 1.** PCA of sperm motility parameters. Loadings, percentage of variance explained and eigenvalues are provided for two main PCs. Major loadings (eigenvectors > 0.3) are in italics.

motility parameters	PC1	PC2
ALH	−0.120	*−0.503*
BCF	−0.030	*0.516*
LIN	*0.460*	0.246
STR	0.191	*0.471*
VAP	*0.452*	−0.278
VCL	*0.325*	−0.290
VSL	*0.504*	0.086
WOB	*0.416*	−0.172
percentage of variance explained	45.11	37.53
eigenvalue	3.61	3.00

ALH, amplitude of lateral head placement; BCF, beat-cross frequency; LIN, path linearity; STR, path straightness; VAP, average path velocity; VCL, curvilinear velocity; VSL, straight-line velocity; WOB, wobble/a measure of path oscillation.

#### (ii) Effects of ejaculate age on sperm motility in seawater and in female reproductive fluid

To test for an interaction between ejaculate age and environmental treatment (seawater versus FRF), we used PC1 and PC2 as response variables in linear mixed models (LMMs) in the ‘lme4’ package of R [67]. CASA also calculates the percentage of motile sperm (total sperm motility), which we used as a response variable in a generalized linear mixed model (GLMM) in the package ‘glmmTMB’ [68]. Total motility was analysed as a proportion bounded by 0 and 1, using a beta distribution and logit link function. All models included ejaculate age, treatment, and their interaction as fixed effects. Models also included random intercept terms for male ID and block ID, and random slope terms that allowed the effects of ejaculate age and treatment to vary among males. Ejaculate age was standardized to a mean of 0 and standard deviation of 1 to improve model stability [69]. LMMs were fit using restricted maximum likelihood (REML). Significance of fixed effects was determined using Type III Wald *χ*^2^ tests for GLMMs, and Wald F tests with Kenward–Roger approximation of degrees of freedom for LMMs [70,71].

Broadcast spawner sperm experience complex patterns of selection, which often include quadratic relationships among traits, fitness and the environment (e.g. [48,50–52,72]). We therefore determined whether a fixed quadratic parameter for ejaculate age should be included in our models using Akaike's Information Criterion corrected for small sample sizes (AICc; [73]). Using the package ‘bbmle’ [74], we compared models with or without a quadratic parameter for ejaculate age (all other effects as described above) and used a minimum reduction in AICc (ΔAICc) of 6 to determine whether the parameter improved model fit [69,75]. If the AICc values supported the inclusion of a quadratic term, we also tested the inclusion of a quadratic age-by-treatment interaction term.

#### (iii) Among-male variation in ageing effects on sperm motility

To test for among-individual variation in the effects of ejaculate age on sperm motility, we separated the data into the two treatment groups (seawater or FRF). Within each of these treatments, we fit LMMs for PC1 and PC2, and GLMMs for total motility, with the same fixed effects structures as described above, a random intercept term for block ID, and either: (i) no random effect for male ID, (ii) a random intercept term for male ID or (iii) a random slope term that allowed the effect of ejaculate age to vary with male ID. We used likelihood ratio tests (LRTs) to compare the reduced models to the full models and determine the significance of the random effects.

#### (iv) Sperm longevity trade-offs and their effect on sperm responses to female reproductive fluid

We used the intercept–slope correlation coefficient from the full PC1 LMM (which is predominantly loaded by velocity and linearity traits; see §3) in seawater as an indicator of a trade-off between initial energy expenditure (i.e. initial velocity and linearity), and longevity. Specifically, such a trade-off would be implied by a negative correlation between initial PC1 values (intercept) and the slope of PC1 with ejaculate age. For the latter, shallower slopes indicate smaller changes with age. We used an LRT to compare the full model with a model with uncorrelated random intercepts and slopes to test the significance of the intercept–slope correlation.

Finally, we tested whether sperm longevity in seawater influenced sperm responses to FRF. To do this, we tested whether the steepness of the ejaculate age-related decline in PC1 in seawater was related to subsequent sperm motility in response to egg-derived FRF at 0, 1, 2 and 3 h post-dilution. We extracted the random intercept and slope coefficients from the model explaining PC1 in seawater. The random intercept values represent the initial scores for PC1, and the random slope coefficients describe the change in this parameter over time in seawater. We used the intercept and slope values as fixed effects in four LMMs that predicted PC1 when sperm were added to FRF at the four post-dilution timepoints. Upon visual inspection of the data, one male was identified as a possible influential datapoint for this analysis at 0 h post-dilution, and another at 3 h post-dilution. We removed these males from the dataset and subsequently ran these models with *n* = 41 males. These models each included a random intercept term for block ID, and the significance of fixed effects was determined as above. Additionally, we ran these same analyses (both the intercept–slope correlation analysis and the trade-off models) for three individual velocity traits (VSL, VAP, and VCL) to assess these relationships for velocity traits specifically. The results were again qualitatively similar to the results for PC1 and support our broad conclusions (see electronic supplementary material for results and brief discussion).

## 3. Results

### (a) Principal component analysis on sperm motility traits

The PCA identified two PCs with eigenvectors greater than 1 (PC1 and PC2). PC1 was predominantly loaded positively by three sperm velocity parameters (VCL, VSL and VAP), WOB and LIN (table 1), while PC2 was predominantly loaded negatively by ALH, and positively by BCF and STR (table 1). In other words, PC1 can be interpreted as a composite measure of directional velocity, with higher scores indicating faster, straighter-swimming sperm, and PC2 can be interpreted as a composite measure of path smoothness, with high scores indicating less deviation of the sperm's curvilinear path from the average path.

### (b) Effects of ejaculate age on sperm motility in seawater and in female reproductive fluid

Including a quadratic parameter for ejaculate age did not improve the fit of the PC1 model (AICc without quadratic parameter: 1176.4; AICc with quadratic parameter: 1181.5; ΔAICc: 5.2) or the total motility model (AICc without quadratic parameter: 461.5; AICc with quadratic parameter: 470.3; ΔAICc: 5.5). Conversely, the quadratic parameter for ejaculate age improved the fit of the PC2 model and was included in the final model for this variable (AICc without quadratic parameter: 1245.0; AICc with quadratic parameter: 1204.1; ΔAICc: −40.9). We compared this quadratic model with a model that incorporated a quadratic age-by-treatment interaction and found that the interaction term did not improve the model's explanatory power (quadratic with interaction: 1204.6, ΔAICc: 0.5). We therefore include a quadratic term for ejaculate age in our final PC2 model, but not in the quadratic age-by-treatment interaction.

**Figure 1.**
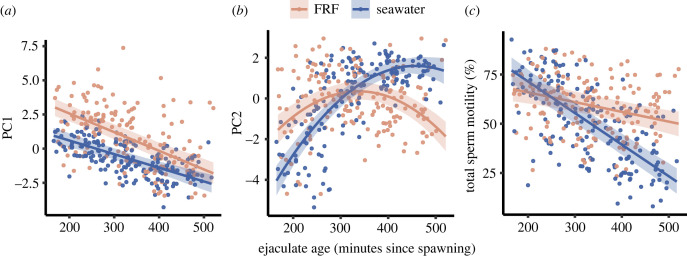
The effect of ejaculate age on (*a*) PC1 (loaded by velocity and linearity traits), (*b*) PC2 (loaded by path smoothness and straightness traits) and (*c*) total sperm motility when sperm motility was measured in egg-derived FRF (pink) or filtered seawater (blue). Shaded areas represent bootstrapped 95% confidence intervals. *n* = 43 males.

The PC1 LMM revealed significant main effects of ejaculate age, treatment and an interaction between the two factors. PC1 decreased with ejaculate age in both treatments, and sperm swam more quickly and in straighter trajectories overall in FRF than in seawater (table 2, figure 1*a*; see electronic supplementary material, table S3 for random effect variance estimates). However, the PC1 slopes differed between treatments, with the decrease in PC1 responses to FRF over time greater than the baseline decline of PC1 in seawater (figure 1*a*).

**Table 2.** Results of LMMs explaining (a) PC1, (b) PC2 and a GLMM explaining (c) total sperm motility. Significance of fixed effects was determined using Wald *Χ*^2^ tests for total sperm motility and Wald F tests and Kenward–Roger degrees of freedom for PC1 and PC2. The random effect structures include random intercepts of male ID and block ID, and among-male slopes by ejaculate age and treatment (seawater or egg-derived FRF). PC1 is loaded strongly by velocity and linearity traits, and PC2 is loaded strongly by path smoothness and straightness traits. Significant *p*-values are in italics. *n* = 43 males.

predictors	estimates	s.e.	f/*χ*^2^ (d.f. = 1)	*p*
(a) PC1				
ejaculate age	−1.24	0.12	110.86	*<0.001*
treatment	−1.49	0.18	65.47	*<0.001*
ejaculate age × treatment	0.33	0.12	7.47	*0.006*
(b) PC2				
ejaculate age	0.06	0.11	0.28	0.596
treatment	0.22	0.20	1.18	0.278
ejaculate age^2^	−0.54	0.07	55.49	*<0.001*
ejaculate age × treatment	1.44	0.13	124.13	*<0.001*
(c) total sperm motility				
ejaculate age	−0.19	0.04	20.62	*<0.001*
treatment	−0.38	0.07	27.39	*<0.001*
ejaculate age × treatment	−0.43	0.05	80.04	*<0.001*

Models were fit with REML. Estimates of variance associated with random effects reported in the electronic supplementary material, table S3.

The PC2 LMM identified a significant interaction between ejaculate age and treatment, and a significant negative quadratic effect of ejaculate age (table 2; see electronic supplementary material, table S3 for random effect variance estimates). Younger ejaculates had lower path smoothness (i.e. greater deviations from average sperm paths) in seawater than in FRF. This pattern switched when ejaculates were older, with PC2 values higher in seawater and lower when responding to FRF (figure 1*b*).

The GLMM for total motility revealed significant main effects of ejaculate age and treatment, and an interaction between these two factors. Total motility decreased with ejaculate age, but in this case the decline in motility at each timepoint in seawater was somewhat alleviated when sperm were added to FRF (table 2, figure 1*c*; see electronic supplementary material, table S3 for random effect variance estimates).

### (c) Among-male variation in ageing effects on sperm motility

For PC1 in seawater, the fit of the full random slope model was not significantly different from either the random intercept only model, or the model without random effects for male ID (table 3*a*). Conversely, a full random slope model was a significantly better fit for predicting PC1 in FRF than both the random intercept only model and the model without random effects for male ID (table 3*b*). Thus, significant among-male variation in ageing effects on PC1 was only detectable when sperm were tested in FRF (see electronic supplementary material, figure S2 for individual reaction norms).

**Table 3.** Results of log-LRTs used to assess significance of random effect terms in LMMs predicting (*a*) PC1 and (*b*) PC2, and GLMMs for (*c*) total sperm motility in seawater and or egg-derived FRF. Reduced models are tested against the full model in each treatment. All models contain a fixed effect of ejaculate age, and a random intercept for block ID. The PC2 model also contains a quadratic parameter for ejaculate age. PC1 is loaded strongly by velocity and linearity traits, and PC2 is loaded strongly by path smoothness and straightness traits. Significant *p*-values are in italics. *n* = 43 males.

	seawater					FRF				
model	log-likelihood	AICc	*G*^2^	DF	*p*	log-likelihood	AICc	*G*^2^	DF	*p*
(*a*) PC1										
male ID random slope (full model)	–232.09	483.94				–326.71	670.07			
male ID random intercept only	–234.70	485.00	5.22	2	0.147	–335.80	684.71	18.18	2	*<0.001*
no random effect for male ID	–236.07	485.54	7.97	3	0.093	–343.13	696.95	32.84	3	*<0.001*
(*b*) PC2										
male ID random slope (full model)	–265.61	556.50				–296.83	616.33			
male ID random intercept only	–277.82	574.91	24.43	2	*<0.001*	–303.02	624.80	12.38	2	*0.004*
no random effect for male ID	–278.69	574.34	26.15	3	*<0.001*	–305.57	627.46	17.47	3	*0.001*
(*c*) total sperm motility										
male ID random slope (full model)	105.71	–196.74				122.83	–230.98			
male ID rand–m intercept only	103.93	–197.51	3.56	2	0.338	121.15	–231.94	3.36	2	0.373
no random effect for male ID	86.36	–164.48	38.71	3	*<0.001*	102.21	–196.19	41.23	3	*<0.001*

The likelihood ratio statistic (*G*^2^) is calculated as −2 × the difference in log-likelihoods between the full model and either the random intercept only or no random effect (of male ID) models. *p* is estimated by comparing *G*^2^ to a *χ*^2^ distribution. Akaike Information Criteria corrected for small sample size (AICc) are provided. *p*-values are adjusted using Bonferroni correction.

The full random slope models explaining PC2 in both seawater and FRF had significantly better fits than the random intercept only models and the models without random effects for male ID, indicating significant among-male variation in the effect of ejaculate age on PC2 in both contexts (table 3*a*,*b*; electronic supplementary material, figure S3).

The fit of the random slope model for total motility was not significantly different from the random intercept only model in seawater; however, the random slope model for total motility was a significantly better fit than the model without male ID random effects (table 3*a*). The same pattern was found in among-male variation of total motility in FRF (table 3*b*). Thus, in both treatments, most of the among-male variation in total motility was accounted for by differences in intercepts, rather than slopes (electronic supplementary material, figure S4).

### (d) Sperm longevity trade-offs and their effect on sperm responses to female reproductive fluid

There was a positive correlation coefficient between random slopes and intercepts in the model of PC1 in seawater (*ρ*_01_ = 0.79; full model summary in the electronic supplementary material, table S4), although this effect was marginally non-significant (LRT: *χ*^2^ = 3.44, d.f. = 1, *p* = 0.064).

**Figure 2.**
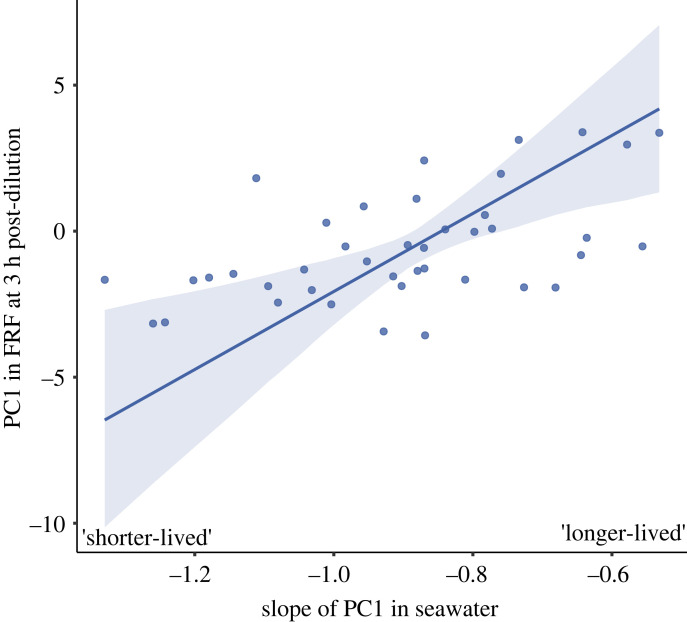
The positive relationship between the slope of PC1 over time in seawater (i.e. sperm longevity) and the predicted value of PC1 in egg-derived FRF at 3 h post-dilution. Slope values to the left of the *x*-axis represent steeper declines in PC1 in seawater, i.e. ‘shorter-lived’ ejaculates. Slope values to the right of the *x*-axis represent shallower declines in PC1 in seawater, i.e. ‘longer-lived’ ejaculates. The figure illustrates that longer-lived ejaculates have greater PC1 values, i.e. greater velocity and linearity, when tested with FRF after being diluted in seawater for 3 h. Shaded areas represent bootstrapped 95% confidence intervals. Plot depicts relationship while holding other predictor variables at their median value. Datapoints represent observed values for *n* = 41 males.

Initial PC1 values in seawater (the random intercept term) did not significantly predict PC1 in FRF at any of the different post-dilution ages (table 4; see electronic supplementary material, table S5 for full model summary). However, changes in PC1 with ejaculate age in seawater (the random slope term) significantly explained PC1 in FRF for the oldest ejaculates, but not for the youngest or intermediate ages (table 4; see electronic supplementary material, table S5 for full model summary). Specifically, steeper declines in PC1 in seawater predicted low PC1 values when old ejaculates were added to FRF, and *vice versa* (table 4, figure 2).

**Table 4.** Results of LMMs explaining PC1 in egg-derived reproductive fluids (FRF) at 0, 1, 2 and 3 h post-dilution. The fixed effects included in each model are the male-specific random intercepts and slopes of PC1 over time in seawater. Significance of fixed effects was determined using Wald F tests and Kenward–Roger degrees of freedom. Significant *p*-values are in italics. A random effect for block ID was included in the models. *n* = 41 males.

time of analysis in FRF (hours post-dilution)	predictor (values from seawater model)	estimates	s.e.	F	d.f.	*p*
0 h	intercept	5.15	3.18	2.62	37.88	0.114
	slope	−3.88	4.33	0.80	38.84	0.376
1 h	intercept	1.29	3.36	0.15	38.64	0.704
	slope	0.46	4.51	0.01	37.28	0.919
2 h	intercept	−0.73	3.39	0.05	37.60	0.831
	slope	4.02	4.64	0.75	38.98	0.392
3 h	intercept	−6.27	3.16	3.94	36.93	0.055
	slope	13.36	4.31	9.63	37.78	*0.004*

Models were fit with REML. Estimates of random effects variance components reported in the electronic supplementary material, table S5.

## 4. Discussion

Our investigation reveals new insights into the key role that FRF play in mitigating ageing effects on sperm motility in external fertilizers and provides novel evidence that patterns of variation in sperm longevity are only revealed when sperm encounter FRF. Three key findings arise from these analyses. First, we found that the effect of ejaculate age on sperm motility depended on the presence of egg-derived FRF, and that FRF can alleviate age-related declines in sperm motility. Second, ageing effects on sperm motility traits varied significantly among males, with stronger variation when sperm were tested in FRF. Third, our findings reveal that greater sperm longevity in the absence of FRF provides the benefit of enhanced motility in response to FRF after extensive periods of ageing—a finding that suggests the relationship between sperm motility and longevity is dependent on the effects of FRF. We discuss the implications of each of these findings below. Collectively, they indicate that to fully understand sperm ageing and phenotypic plasticity, we need to consider interactions between sperm and FRF.

The significant interactive effects of treatment and ejaculate age on PC1 (loaded by velocity and linearity traits) and total motility demonstrate that ageing effects on sperm motility are dependent on FRF. Total sperm motility declined quickly in seawater as ejaculates aged, but this decline was alleviated to some extent when FRF was added to old ejaculates, which suggests some capacity of FRF to ‘rescue’ sperm from potentially adverse reductions in motility over time (see also [12,19,39,47]). This could indicate that as sperm age, they retain some ability to be ‘activated’ by FRF, despite decreased motility in seawater (e.g. [17]). Alternatively, sperm might adaptively reduce swimming activity over time in seawater to conserve energy before they encounter egg cues [47,48]. In contrast with total motility, PC1 in FRF declined more quickly with ejaculate age than PC1 in seawater. Thus, although FRF increased the proportion of motile sperm for old ejaculates, the individual sperm of older ejaculates appear to have weaker responses to FRF (i.e. lower velocity and path linearity) than those of fresh ejaculates. This could indicate diminishing beneficial effects of FRF over time for individual sperm, although this may depend on the concentration of FRF, which was not varied in our study. An interesting next step could be to explore how FRF concentration potentially alters these patterns.

PC2 differed significantly between seawater and FRF and exhibited a nonlinear relationship with ejaculate age. Younger ejaculates had lower PC2 values in seawater than they did in response to FRF. Low PC2 values correspond to less frequent flagella beating, greater amplitude of head movements and more curved path trajectories. Curved swimming patterns of broadcast spawner sperm in seawater are thought to represent a ‘searching’ behaviour that increases the chances of sperm encountering eggs or FRF [48,50]. Hence, we may expect sperm to exhibit greater deviations from their average path in seawater as they attempt to locate FRF. By contrast, the higher PC2 values in FRF, which were generally maintained over the course of the experiment, suggest more frequent flagella beating with less head movement and a straighter path trajectory, which is suggestive of a chemotactic response (see also [21]). This pattern changed for older ejaculates, with greater PC2 values, i.e. smoother paths, in seawater than in FRF. This may indicate that older sperm have a reduced ability to maintain effective (curved) search patterns in seawater. To our knowledge, the energetic costs to sperm of maintaining particular swimming paths over time have received no attention (compared to sperm velocity; e.g. [76]). Further tests of this idea across multiple environments would be helpful for understanding functional effects of sperm ageing.

We found significant variation among males in the way ejaculate age affects sperm behaviour, and for PC1 in particular, such variation was more pronounced when sperm were added to FRF than in seawater. This implies that while ageing in seawater affected PC1 values in similar ways among males, only certain males were able to maintain fast, linear responses to FRF as their sperm aged. Therefore, there may be more opportunity for selection to act on variation in how sperm respond to FRF as they age, than variation in how sperm swim in seawater as they age. While such predictions need to be confirmed by measurement of relative fitness (e.g. fertilization success) of sperm in the different conditions, previous findings in *M. galloprovincialis* provide strong support that selection favours males whose sperm respond strongly to FRF [21,50,52]. Another possibility is that the generally consistent age-related motility declines in seawater among males may reflect historical selection to conserve energy until sperm locate chemical cues. Future studies could usefully explore the adaptive outcomes of the interaction between FRF and sperm ageing to test between these scenarios.

We show that sperm with greater longevity had stronger responses to FRF after periods of ageing but found no evidence for a motility–longevity trade-off in seawater only (i.e. in the absence of FRF). Specifically, sperm that exhibited greater longevity in seawater over the experimental timeframe (as measured by changes in PC1 with ejaculate age) responded to FRF with higher PC1 values, i.e. greater velocity and linearity, at the oldest post-ejaculation timepoint. This may suggest that stronger responses to FRF are advantageous shortly after spawning [32,47,48], but males with longer-lived ejaculates may have an added advantage when there is a longer time between spawning and fertilization. Although we did not measure fertilization success directly, prior work has consistently found that in *M. galloprovincialis*, slow-swimming sperm have a fertilization advantage under gamete limitation (when gamete encounters are rare) and faster sperm have higher success at high gamete abundance [48,51,52]. The present findings suggest that these fitness advantages could be linked to variation in the time lag between spawning and encountering FRF. A trade-off between sperm velocity and longevity has been reported in at least one other broadcast spawning invertebrate [32], but the evidence more generally for such trade-offs is inconsistent across many studies and taxa (e.g. presence of trade-off: [33–35]; absence of trade-off: [38,77,78]). Importantly, however, such trade-offs have typically been examined in the absence of FRF. Our findings suggest that incorporating sperm responses to FRF could clarify the extent of sperm motility–ageing trade-offs across mating systems.

## 5. Conclusion

Our study provides novel insight into the effects of sperm ageing and FRF on sperm motility in an external fertilizer. Our findings allow us to draw three broad conclusions about the effects of egg-derived FRF on sperm and highlight the value of considering plasticity in sperm motility across environmental conditions. First, we find that declines in sperm motility traits over time were lessened by FRF, a finding that supports recent commentary around the importance of FRF for sperm function ([10]; see also, e.g. [23]). Second, we found significant among-male variation in ageing effects on sperm motility, but particularly in the responses of sperm to FRF as they aged. This finding highlights the importance of FRF in understanding the potential adaptive outcomes of sperm longevity. Finally, we found evidence for a relationship between sperm longevity and sperm behaviour that was only revealed when sperm were tested in the presence of FRF. This latter finding is suggestive of an advantage for sperm that can sustain energy stores and maintain effective responses to egg-derived FRF when there is a lag between spawning and fertilization. Overall, our study emphasizes the value of exploring female effects in the context of sperm ageing and phenotypic plasticity, as doing so may expose important variation among males and environments in sperm functional traits.

## Data Availability

Data and code associated with this manuscript are available from the Dryad Digital Repository [79]. Supplementary material is available online [80].
